# Organic bioelectronics probing conformational changes in surface confined proteins

**DOI:** 10.1038/srep28085

**Published:** 2016-06-17

**Authors:** Eleonora Macchia, Domenico Alberga, Kyriaki Manoli, Giuseppe F. Mangiatordi, Maria Magliulo, Gerardo Palazzo, Francesco Giordano, Gianluca Lattanzi, Luisa Torsi

**Affiliations:** 1 Dipartimento di Chimica, Università degli Studi di Bari *Aldo Moro* - Bari (Italy); 2 Dipartimento Interateneo di Fisica “M. Merlin” dell’Università e del Politecnico di Bari - Bari (Italy); 3 Dipartimento di Farmacia – Scienze del Farmaco, Università degli Studi di Bari *Aldo Moro* - Bari (Italy); 4 Dipartimento di Medicina Clinica e Sperimentale –Università degli Studi di Foggia - Foggia (Italy)

## Abstract

The study of proteins confined on a surface has attracted a great deal of attention due to its relevance in the development of bio-systems for laboratory and clinical settings. In this respect, organic bio-electronic platforms can be used as tools to achieve a deeper understanding of the processes involving protein interfaces. In this work, biotin-binding proteins have been integrated in two different organic thin-film transistor (TFT) configurations to separately address the changes occurring in the protein-ligand complex morphology and dipole moment. This has been achieved by decoupling the output current change upon binding, taken as the transducing signal, into its component figures of merit. In particular, the threshold voltage is related to the protein dipole moment, while the field-effect mobility is associated with conformational changes occurring in the proteins of the layer when ligand binding occurs. Molecular Dynamics simulations on the whole avidin tetramer in presence and absence of ligands were carried out, to evaluate how the tight interactions with the ligand affect the protein dipole moment and the conformation of the loops surrounding the binding pocket. These simulations allow assembling a rather complete picture of the studied interaction processes and support the interpretation of the experimental results.

The study of proteins confined on a surface is of great relevance as they are routinely being used in several key relevant biological measurements in laboratory and clinical assays, such as enzyme-linked immunosorbent (ELISA)^1^ and surface plasmon resonance (SPR)^2^ assays as well as Quartz-Crystal Microbalance (QCM)^3^ ELISA is a label-needing technology while SPR is regarded as a gold standard in label-free analytical bio-assay being among the most powerful methods involving surface-anchored biomolecules. It is a label-free technique that can achieve detection limits in the nanomolar range. Atomic Force Microscopy, operated in Force Spectroscopy mode can measure the force, energy and length scale of biorecognition events and can map the presence of recognition groups on the surface. Beautiful examples of how very interesting insights can be gained with this approach, also in combination with organic bioelectronic devices have been recently produced^4^. However, in general it requires a rather large statistic and data analysis of force curves that is only partially automated. QCM is also label-free, quantitative and ultrasensitive but a rather indirect analysis of the output features is generally needed. Poorly reproducible are also the biosensing methods involving nanoscopic and low dimensional field-effect transistors (FETs)^5^.

Often, the confinement of proteins or peptides on a surface results in the aggregation of structures that may be characterized by different degrees of order. However, it is quite difficult to control the aggregation processes, and to understand how the molecular details impact on and/or determine the macroscopic structure and its relative mechanical and electric properties. A thorough understanding of the self-assembly of highly ordered peptides or biomolecules has, in general, a twofold purpose: (*i*) it can offer insights into the onset of pathological diseases where protein aggregation plays a crucial role (e.g. neurodegenerative diseases)^6^; (*ii*) it provides valuable information for the bottom-up design and fabrication of nanoscale devices and sensors for biomedical applications^7^. In this respect, organic bioelectronics can be a precious tool to achieve a deeper and more complete understanding of the processes involving protein interfaces, since the energetic and electrostatic contributions play the main role in shaping the response of its devices^8^,^9^,^10^,^11^.

Biotin-binding proteins such as avidin (AV), streptavidin (SAV) and neutravidin (NAV) have been widely studied as prototype systems to investigate binding-induced conformational changes in biological macromolecules^11^. They share a number of features, the most relevant one being their extraordinary high binding affinity to the biotin molecule (vitamin B_7_). As an instance, the AV-biotin dissociation constant (K_d_) in solution falls in the femto molar (fM) range^12^. The three proteins share the same tetrameric structure characterized by four identical monomeric subunits each endowed with a β-barrel pocket to host a biotin molecule. Differences are mostly related to their post-translational modifications. In fact, AV is a glycosylated protein exhibiting a basic isoelectric point (pI) of *ca.* 10^13^. SAV is a non-glycosylated protein with a slightly acidic pI ranging between 5 and 6^11^, presenting functional domains similar to AV, with about 33% identical residues^13^. NAV is a non-natural de-glycosylated form of AV with a pI of 6.3^11^.

Several methods can be used to investigate the changes occurring in protein tertiary and quaternary structures upon binding. Most of the approaches have been tailored to study proteins dispersed in bulk solutions whose properties are investigated by means of fluorescence, circular dichroism or Fourier-transform infrared spectroscopy^14^,^15^. At variance, crystallized proteins can be studied by means of X-ray diffraction methods^16^,^17^,^18^. The crystallographic data available for these proteins suggest that each S(AV) monomer consists of a long chain that loops around the bound biotin^19^. The conformation of these loops is largely dependent on the presence of biotin, with biotin binding reducing the fluctuations of the surrounding residues. Moreover, although biotin binding is essentially non-cooperative^20^, a more entangled structure involving S(AV) sub-units has been already postulated based on the experimental evidence that biotin binding in solution increases the protein denaturation temperature in excess of about 30 °C^21^. A ^1^H/^2^H exchange study further supports this hypothesis^19^. In addition, the binding process induces a conformational change of the loops surrounding the β-barrel cavity, switching from an *open* to a *closed* state that clamps the bound biotin^22^.

Despite the broad range of studies involving biotin complexes, the binding energetics and the dynamics of the process are not fully understood yet^10^. For instance, changes in the electrostatic and polarization properties of these proteins upon binding are still poorly experimentally investigated. On the other hand, computational studies have recently predicted that the electrostatic polarization of biotin-binding proteins provides a substantial contribution to the free energy of its extraordinary strong binding process^23^. This is ascribed to the stabilization of the hydrogen bond between the biotin and the tyrosine moiety while the electrostatic interaction between biotin and the nearby residues appears to be responsible for the enhanced binding affinity. Further modeling studies, employing quantum mechanics/molecular mechanics simulations were performed on biotin binding to SAV^24^.

Lately, several studies have involved also thin-films or self-assembled monolayers of proteins deposited on a solid surface^25^,^26^. These studies are mostly carried out by means of Surface Plasmon Resonance (SPR)^27^, Surface-Enhanced Raman Scattering (SERS)^28^.and Atomic Force Microscopy (AFM) with bio-functionalized AFM tips^29^,^30^. SERS and AFM approaches have been proposed only more recently and are, as yet, still affected by rather poor reproducibility. Moreover, SERS studies are generally limited to aromatic amino acids and small peptides^31^. Notably, none of the elicited approaches can provide experimental information on the electrostatic component of the interaction energetics, which has been predicted to be one of the main players in the protein-biotin interaction process.

Organic thin-film transistors^32^,^33^ have been proved to be very powerful in studying processes occurring at biological interfaces^8^,^10^,^34^,^35^,^36^,^37^. One of the main advantages of this approach is the possibility to address key information by measuring the device multiple figures of merit^38^. Recently, it has been shown that organic thin-film transistors (OTFTs) allow the accurate determination of the free-energy balances associated with a binding process. In turn, these energetic components (including the electrostatic one), as well as the dynamics involved in conformational changes, have been quantified^39^. In the last years, different TFT configurations have been proposed for biosensing applications. One of them is produced by directly interfacing the OSC with an electrolyte solution. This has been achieved by means of the the electrochemical transistors^40^ and the electrolyte gated TFT^41^. Moreover, very sensitive determinations become possible when the direct interfacing of a biological recognition layer with a transistor electronic channel is exploited^42^. This has been achieved with the functional bio-interlayer thin film transistors (FBI-OTFTs)^43^, whose detection limits have been proved to reach the pM range. This device configuration has allowed achieving unprecedented performance level in terms of selectivity, accuracy and reproducibility as well. On the other hand, the morphology of the organic semiconductor is of great relevance to obtain high electronic performances, particularly as far as the field-effect mobility and on/off ratio are concerned. It is, thus, crucial to assess the FBI-OTFT fabrication protocol leading to a stable bio-recognition layer/organic semiconductor interface. More importantly, it is critical that the properties and the quality of the dielectric/organic-semiconductor interface are controlled as they also strongly impact on the device performance and stability. The morphology and the structure of the stacked bilayer formed by the protein bio-interlayer and the overlying organic semiconductor has been recently investigated for different protein deposition methods, such as Layer by Layer and spin coating^37^. This study has combined X-ray scattering techniques and scanning electron microscopy to gather relevant information on the interface structure down to the nanoscale and to assess target analyte molecules capability to percolate through the semiconducting layer reaching the protein deposit lying underneath.

In this work, FBI- and the newly introduced pre-formed complex (PFC)-OTFTs are proposed as devices capable to accurately probe biotin-biding events occurring at a protein interface. Both devices embed a layer of SAV, NAV or AV that resides right at the dielectric/organic-semiconductor (OSC) interface. The output current change upon binding, taken as the transducing signal^8^, is further decoupled into its component figures of merit. This procedure allows to separately addressing key features of the protein-ligand interaction considering both electrostatic and conformational changes. All-atoms MD simulations were performed on the whole AV tetramer with and without ligands. This provides a molecular rationale for the hypotheses that are needed to interpret the experimental findings.

## Results

**Figure 1:**
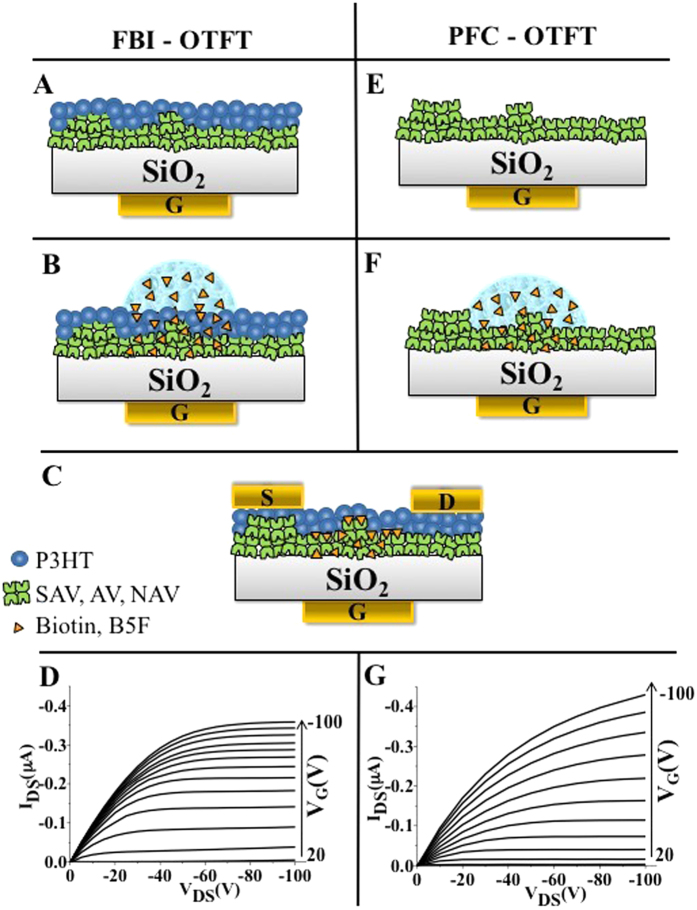
FBI and PFC device fabrication steps and output I–V characteristics. Schematic illustrations of the fabrication steps implemented for the realization of the functional bio-interlayer (FBI) OTFT (**A–C**) and a pre-formed complex (PFC) OTFT (**E,F,C**). P3HT is reported in blue, SAV, AV or NAV layer is shown in green, while the orange triangles represent biotin or B5F. See text and method session for details. I–V characteristics (I_DS_ *vs.* V_DS_ measured for V_G_ values ranging between +20 V to −100 V in steps of −10 V) for the FBI (**D**) and PFC (**G**) structures respectively. The devices integrate an AV layer and have been exposed to 1 ppb of Biotin.

The most relevant steps in the fabrication of an FBI-OTFT are illustrated in Fig. 1A–C. The FBI layers are deposited by very low speed spin-coating from a water solution, on a SiO_2_ surface, resulting in an island-like structure, with features that are inhomogeneous in size and distributions as recently proven by Scanning Electron Microscopy (SEM) characterization^37^. Subsequently, a regioregular poly-3-hexylthiophene (P3HT) p-type OSC is deposited by spin-coating from chloroform on top of the FBI layers (Fig. 1A), smoothly covering the protein clusters. It has been proven that the P3HT forms a layer full of hollows and voids, covering the protein cluster^37^. Finally, a row of source (S) and drain (D) contact pads is patterned on top of the OSC. The device is exposed to a fixed aliquot of 1 part-per-billion (1 ppb, 5 nM) biotin solution (Fig. 1B) and, after incubation, the unreacted biotin is removed by washing with water (Fig. 1C). Notably, the porous nature of the OSC allows biotin molecules to efficiently diffuse through the OSC layer and thus reach the proteins deposited underneath^33^,^37^. The PFC-OTFT configuration fabrication steps are shown in Fig. 1E,F,C. Also in this case the biotin-binding proteins are deposited by slow spin-coating on the SiO_2_ surface (Fig. 1E). At variance with the FBI-OTFT configuration, exposure to biotin, and eventually complex formation, is achieved before OSC deposition. To this aim, two deposits of protein are placed on two different locations in the same SiO_2_ substrate: one is exposed to water and the other to the 1 ppb biotin solution. The same aliquots of solution as for the case of the FBI-OTFTs are used. After incubation, the unreacted biotin is washed away. For the sake of clearness, in Fig. 1F only the region of the protein exposed to biotin is represented. The last step consists in the deposition of P3HT on the layer of pre-formed complexes along with the row of S and D contact pads placed on top of the OSC (Fig. 1C).

**Figure 2:**
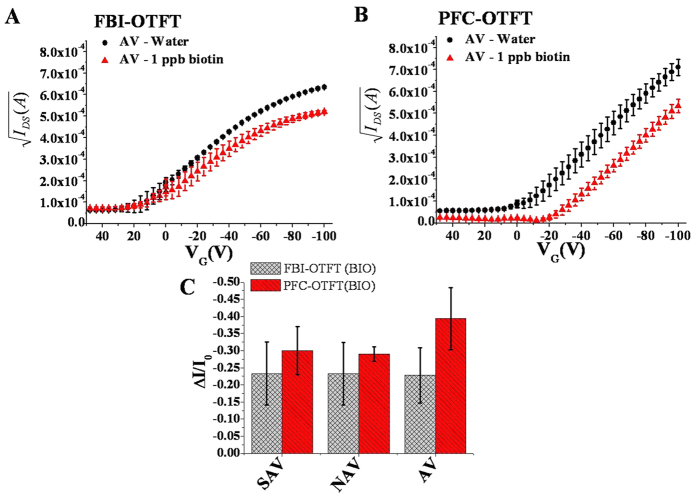
FBI and PFC I–V transfer characteristics. Square root of the source-drain channel current *vs.* V_G_ for AV embedding OTFTs when exposed to water and to 1 ppb of Biotin. Data relative to (**A**) FBI-OTFT and (**B**) PFC-OTFT, respectively. Data are relevant to three repeated measurements and are reported as the average value along with the relevant RSDs. (**C**) Normalized current response ΔI/I_0_ for AV embedding FBI and PFC-OFET upon exposure to 1 ppb of Biotin. Data are relevant to an ensemble of measurements acquired on nine different devices (reproducibility error) and are reported as the average value along with the relevant RSDs.

Typical I–V output characteristics (source-drain current, I_DS_ *vs.* source-drain voltage, V_DS_ with the gate voltage, V_G_ ranging from +20 V to −100 V in steps of −10 V) measured for the FBI and PFC OTFTs embedding AV-biotin complexes, are reported in Fig. 1D,G, respectively. The curves exhibit a good level of current modulation as well as nicely shaped linear and saturated regions along with low leakage currents at low V_DS_. The I_DS_ current flowing in the TFT channel in the saturation regime is given by^43^:


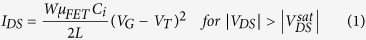


where W and L are the channel width and length, respectively, while C_i_ is the gating system capacitance per unit area. V_T_ is the OTFT threshold voltage while μ_FET_ is the field-effect mobility^37^. According to Eq. 1 √I_DS_ is linear in (V_G_ − V_T_), with V_T_ being the intercept to the V_G_ abscissa, while (C_i_ . μ_FET_) is proportional to the slope of the linear segment of the curve. Typical √I_DS_ *vs.* V_G_ curves for AV embedding FBI and the PFC OTFTs measured after exposure to water and 1 ppb of biotin, are reported in Fig. 2A,B, respectively. Visual inspection of these curves already reveals a change in the slope of the linear region of the curves for the FBI-OTFT devices, while no significant shift along the V_G_ axis can be observed upon binding. The curves measured for the PFC-OTFT show, on the contrary, a fixed slope and a V_T_ shift. A linear interpolation of these curves allows quantifying these changes by extracting the devices figures of merit^44^. Concerning the gating capacitance of the bioelectronic OTFTs under study, it is necessary to clarify the following. C_i_ is the result of three capacitances in series: the capacitance of the SiO_2_ dielectric layer (9 nF cm^−2^), the capacitances of the protein layer and that of the electronic channel in the OSC, the latter two both falling in the μF cm^−2^ range^45^. The slight ionic conductivity of the protein makes the film more conductive and endowed with a larger capacitance than the 300 nm thick SiO_2_^46^. Being these three contributions in series, the resultant C_i_ is dominated by the SiO_2_ dielectric capacitance values, as it is by far the smallest^39^. Clearly, this capacitance will not change upon binding of the ligand to the biolayer. This is a relevant point and eventually it can be inferred that the changes in the I–V curves slope upon binding are ascribable only to changes of the OSC field-effect mobility.

The current-voltage transfer curve (I_DS_, *vs.* V_G_ at V_DS_ = −80 V) is measured before and after the formation of the biotin-protein complex. I_DS_ measured at V_G_ = −100 V after binding is taken as the *signal* value (I). The prior measurement with the same setting but employing a water (biotin-free) solution) provides the *base-line* value (I_0_). The normalized current response is evaluated from the measured transfer characteristics as:


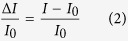


where I and I_0_ are the elicited I_DS_ *signal* and *base-line* values, respectively. The ΔI/I_0_ is taken as the electronic response because it normalizes the device-to-device variation in TFT biosensors^45^, thus allowing a highly reproducible data set. The ΔI/I_0_ fractional decreases (averaged over 9 different devices) are shown in Fig. 2C, turned out to be very similar for the FBI and the PFC OTFTs. In the case of SAV, the values are equal to −0.23 ± 0.01 and −0.30 ± 0.17, respectively. A two-sided t-student test at a level of significance of 0.05 confirms that the two values are indeed not significantly different. Similar conclusions can be drawn also for the device embedding AV and NAV. This evidence demonstrates that, despite the differences in the FBI- and PFC-OTFT preparation procedures, the two classes of devices yield a comparable number of TFT detectable biotin-binding events. Such an occurrence is expected as, from data gathered in a previous study^29^, the percolation of a biotin solution through the OSC in an FBI-OTFT structure is expected to have a yield well in excess of 80%.

Remarkably, while the fractional changes of the current are constant, those of the μ_FET_ and the V_T_ figures of merit, are significantly different for the FBI- and the PFC-OTFTs. These relative changes can be correlated to the I_DS_ fractional changes by differentiating Eq. 1. Assuming C_i_ to be constant, the following holds, up to first order:


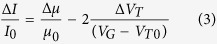


with V_T0_ and μ_0_ being the threshold voltage and the field-effect mobility values extracted from the *base-line* current curve.

**Figure 3:**
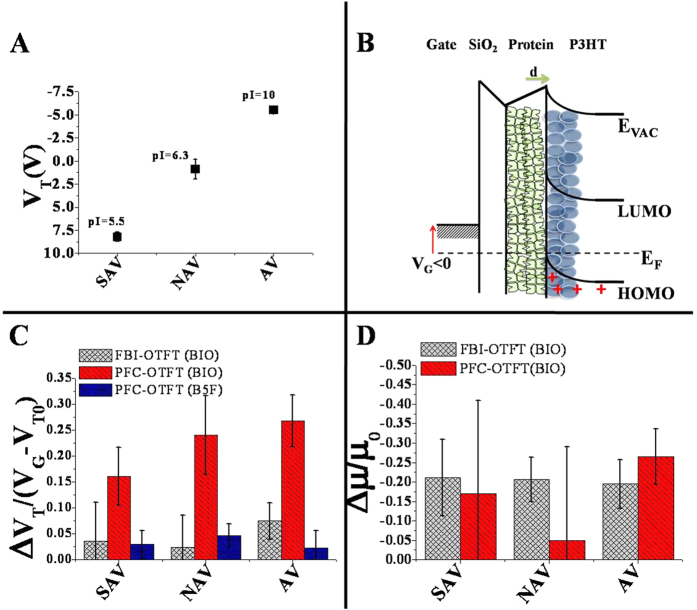
Threshold voltage and field-effect mobility fractional changes. (**A**) Threshold voltage (V_T_) values measured for OTFT devices embedding SAV, NAV and AV. Data are relevant to nine measurements acquired on different devices and are reported as the average value along with the relevant RSDs. (**B**) Band diagram of an OTFT embedding the protein layer underneath the OSC, upon application of a negative gate bias that drives the transistor in the accumulation mode. (**C**) Fractional changes of the threshold voltage evaluated for the FBI configuration upon exposure to 1 ppb of Biotin and for the PFC configuration upon exposure to 1 ppb of Biotin and B5F. (**D**) Fractional changes of the field-effect mobility evaluated for the FBI and PFC configurations upon exposure to 1 ppb of Biotin.

Figure 3A shows the V_T_ values measured for the devices embedding the three proteins. Figure 3B displays a schematic diagram of the energy levels for an OTFT embedding an AV protein layer at the OSC/dielectric interface. V_T_ fractional changes for the FBI- and PFC-OTFTs upon exposure to biotin and biotin (5-fluorescein) conjugated (B5F) are reported in Fig. 3C. Due to the presence of the carboxyl group, bare biotin carries a net negative charge at the experimental pH of 7.5, while in the B5F this site is bounded to fluorescein thus making B5F neutral. Significant V_T_ fractional changes are observed for the PFC-OTFTs, namely: an average V_T_ fractional change of 0.27 ± 0.05 is observed for the AV-biotin complex, 0.24 ± 0.08 results for the complex involving NAV while V_T_ fractional change is 0.16 ± 0.06 in the case of the SAV-biotin complex. Apparently, the ΔV_T_/(V_G_ − V_T0_) values are all positive. The (V_G_ − V_T0_) term is always negative, being the V_T0_ *base-line* threshold voltage values consistently less negative than the V_G_ value at which the fractional changes are evaluated (V_G_ = −100 V). Eventually, it can be stated that all the ΔV_T_ shifts evaluated are negative. The data in Fig. 3C show also that a negligible V_T_ fractional change is measured in the PFC-OTFTs for all the protein-B5F complexes. Strikingly, Fig. 3C shows also how biotin binding has a negligible impact on the V_T_ of the FBI-OTFT devices. Figure 3D compares the fractional decreases of μ_FET_ for FBI and PFC devices. In the FBI configuration, a decrease of 0.20 ± 0.07 is consistently observed for all three proteins. On the contrary, PFC-OTFT devices produce much more scattered μ_FET_ relative changes showing no correlated values for the three proteins upon biotin binding.

**Figure 4:**
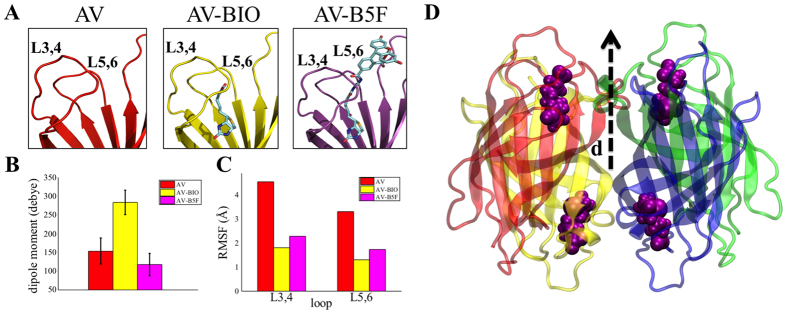
Molecular dynamics simulations of Biotin-binding proteins. (**A**) Residues close to the binding pocket of the AV (left), AV-Biotin complex (center) and Avidin-B5F complex (right). (**B**) Histogram showing the dipole moment (Debye) averaged along the obtained molecular dynamics trajectory for AV (red), AV-Biotin (yellow) and AV-B4F (magenta) systems. A single value is reported for each system averaging statistics from the two monomers. (**C**) Histogram showing the computed Root Mean Square Fluctuations (RMSF) (Å) values for AV (red), AV-BIO (yellow) and AV-B4F (magenta) systems considering both L3,4 and L5,6 loops. For each system, the averaged RMSF, computed from the two monomers, is reported. (**D**) Overall tetrameric structure of the simulated AV with bound biotin molecules. Dipole directions are shown (not to scale).

The structures of AV and SAV were obtained from the Protein Data Bank (PDB) and the protonation state of the residues as well as the charge distributions were accounted for considering the charge associated with the protein residues at the pH of the AV and SAV solutions (7.5). The charge distribution of NAV could not be evaluated since its structure is not available in the PDB. At pH 7.5, arginine and lysine residues carry a positive charge, while glutamate and aspartate ones are negatively charged. To mimic the experimental conditions corresponding to a layer of densely packed dry proteins, MD simulations were performed with an implicit solvent model, assuming a relative permittivity ε_r_ of 3, typical for a protein system^44^. This was applied on the AV tetramer (taken as a model system for the three proteins) to investigate how the tight interactions with biotin and B5F affect the protein dipole moment and the conformation of the loops surrounding the binding pocket^19^. The L3,4 loop (connecting strands *β*3 and *β*4 – residues from position 35 to position 45) and the L5,6 (connecting strand *β*5 and *β*6 – residues from position 70 to position 75) of all the considered systems, are shown in Fig. 4A. In Fig. 4B the magnitude of the electric dipole moments computed for AV as well as for its complexes with biotin and B5F, are shown. A single value averaged along the entire MD trajectory is reported for each considered system. Specifically, the bare AV and the AV-B5F complexes exhibit close dipole moments of 154 ± 35 D and 118 ± 29 D, respectively, while the value computed for the AV-biotin complex is 283 ± 33 D. The time-dependence of the computed dipoles supports the evidence that the AV-biotin holds an associated electric dipole moment that approximately doubles the value found for the system in which the biotin binding pocket is empty. This difference is stable for almost the entire time-span of MD simulations. The Root Mean Square Fluctuations (RMSF) values were computed for the L3,4 and the L5,6 loops. The results, reported in Fig. 4C, clearly show that the molecular flexibility of both loops is strongly limited upon binding of both biotin and B5F. This is actually consistent with reported experimental and computational data showing how an *open* arrangement of L3,4 is not allowed upon biotin binding in AV, while biotin forces L3,4 in a *closed* conformation^14^,^47^. In addition, MD simulations indicate that also the L5,6 has a similar fate. In this case the tendency to adopt a *closed* conformation is even more pronounced. In this respect, the RMSF values and the time-dependent evolutions of the Root Mean Square Deviations (RMSD) values strongly support the hypothesis that the molecular behavior of L3,4 and L5,6 is similarly influenced by biotin and B5F binding.

## Discussion

The conformational changes occurring in proteins immobilized underneath the electronic channel of an OTFT can impact on the electronic properties of the device in a two-fold fashion^39^: (*i*) if the gate electrode or the OSC electrochemical potential is affected, the measured shift in V_T_ is related to a change in the interfacial protein layer electrostatic properties; (*ii*) if the conformational change impacts on the transport properties of the OSC, μ_FET_ is affected by the structural/morphological changes occurring in the protein layer. Interestingly, with the FBI- and the PFC-OTFT configurations, the V_T_ and μ_FET_ contributions to the measured current fractional changes can be decoupled and separately addressed. Hence, the combined use of these two structures allows gaining a rather complete picture of the electrostatic and conformational changes occurring to a protein while it interacts with its affinity ligand. No other available techniques to study thin-films or self-assembled monolayers of proteins deposited on a solid surface are capable to provide these important pieces of information.

Here, we first discuss why V_T_ shifts allow quantifying the electrostatic changes occurring at the OSC/dielectric interface. The capacitance coupling between the SiO_2_ gate-dielectric and the OSC determines the conditions to generate a two-dimensional (2D) electronic channel of free-charges in the OSC^44^,^48^. These charges are injected and eventually drift under the V_DS_ bias. However, although when V_G_ is applied the conditions for the 2D free-charge accumulation at the dielectric/semiconductor interface are set, the actual I_DS_ current flow in the OSC does not start (under a given applied V_DS_) until V_G_ exceeds the transistor V_T_. In a trap-free device, V_T_ is in fact the bias needed to offset the built-in potential generated by the mismatch existing between the metal-gate and the OSC electrons electrochemical potentials or Fermi levels. As V_G_ equals V_T_, the energy barrier is flattened and, when the V_G_ bias exceeds V_T_, further injected charges can drift through the channel with a given μ_FET_^43^.

Of interest to this study is the occurrence that the presence of a protein interlayer between the gate-dielectric and the OSC will determine a further shift of the TFT V_T_ according to the protein electrostatic properties. Similarly, also the formation of the complex between such an immobilized protein and its affinity ligand will cause a V_T_ shift that quantifies the electrostatic properties of the protein as the complex is formed. The protein layer electrostatic properties can be modelled by assuming that the positively and negatively charged residues characterizing the SAV, NAV and AV tetramers are surrounded by the counter-ions coming from the buffer solution used during the protein purification. The charged residues and the associated counter-ions give rise to a dipole moment whose resultant has been evaluated for the whole AV tetramer, taken as a model for the three proteins investigated, in MD simulations with implicit solvent, considering a relative permittivity ε_r_ = 3 (Fig. 4B).

Due to the rather uneven morphology^37^, the overall protein film thickness used in the present study is not uniform, although the estimated protein surface density (10 pM/cm^2^) is compatible with the deposit of a fully packed monolayer^36^. Nonetheless, in the proposed device structures, the uniformity of the protein layer is not critical for the following two main reasons: (*i*) the contribution of its capacitance to the gating system is negligible; (*ii*) only the outermost portion of the protein layer will influence anyhow the electrostatics of the OSC. The latter relies on the assumption that each protein can be represented by a permanent dipole moment that induces a dipole in the OSC (Debye interaction) with the interaction force being a function of d^−6^ 49. Indeed, the screening length can be computed as the cutoff radius of the Debye interaction force whose estimate for the AV/OSC interface is 1.3 nm. Consequently, only a permanent dipole moment residing in the protein at the interface can affect the OSC interfacial region. In a TFT configuration this is the only portion of the OSC film that matters, as it is where the 2D electronic transport takes place. The interface between the *ca.* 3 nm top protein monomeric subunit and the OSC 2D electronic channel (<5 nm) falls within this range. In other words, the TFT sees only the interfacial monolayer of proteins independently from the presence of multilayer protein clusters. As the symmetric nature of the tetrameric biotin-binding proteins is broken by the presence of the interface with the OSC, the protein layer will induce a net dipole moment in the OSC even if the protein layer is not ordered. This happens because the protein layer induces a net dipole moment as the closer monomeric subunits of the protein will have a much stronger interaction with the OSC than the farther off ones. Clearly, the dipole moment orientation in the OSC interfacial layer will have the same orientation as that of the protein or of the complex under investigation. It is also important to remark that the OSC highly conformable nature allows this layer to follow very nicely the unevenness of the cast protein layer, resulting in a large protein/OSC interface.

Data in Fig. 3A show the V_T_ shifts, measured when the SAV, NAV and AV proteins are embedded into the OTFT. The measured V_T_ values do asses that an overall permanent dipole moment can be associated with the protein interfacial layer that, in the case of the SAV, is oriented with its negative pole facing the OSC^50^,^51^, while the opposite direction is observed for the AV as depicted in the schematic model reported in Fig. 3B. As expected, a very weak dipole is associated to NAV. MD simulations performed on the AV tetramer confirm the conclusion that this protein is indeed endowed with an intrinsic permanent dipole moment. Figure 4D displays the AV tetramer and the direction of its dipole moment associated to biotin binding. It is important to compare our results to a recent study performed on an OTFT including a self-assembled monolayer of differently charged quartz-binding polypeptides attached to the SiO_2_ dielectric at the interface with a pentacene OSC^52^. In this work, the direction of the V_T_ shifts are assumed to follow the orientation of the fixed dipoles associated with the polypeptides and the counter-ions distributed as the outermost layer at the interface with the OSC. However, the investigation of the changes in the electrostatic properties of the protein layer upon formation of the protein-biotin complex, to the best of our knowledge, has never been explored before.

The data reported in Fig. 3C quantify the electrostatic contribution to the fractional current change for the SAV, NAV and AV proteins and their complexes with the biotin or B5F ligands. The positive ΔV_T_/(V_G_ − V_T0_) implies that the formation of the pre-formed complexes shift V_T_ towards more negative values. Therefore, upon biotin binding, the induced dipole moment increases in absolute value and is oriented in the direction opposite to the one induced by the polarization field of the gate. This means that the dipole moment of the complex layer points (or has a significant component directed) towards the OSC layer. Moreover, for all proteins, a systematically larger ΔV_T_/(V_G_ − V_T0_) value is observed for the PFC-OTFTs as compared to the FBI-OTFTs. This occurrence can be explained by considering that the formation of the induced dipole orientation in the OSC can be achieved only in the PFC device structure as the binding-induced dipole moment change occurs before the OSC is deposited. Indeed, during the OSC deposition process, the permanent dipole of the cast proteins can induce a net polarization only when the OSC solvent molecules are present. This is in line with published theoretical results, proving how the presence of residual solvent molecules increases the free space available to the polymeric chains, thus allowing a rapid chain reorientation^53^. Under such circumstances the OSC can rearrange its structure to minimize the electrostatic interaction energy with the protein layer deposited beneath. Eventually, the dipole moment induced in the OSC at the interface with the cast proteins will be *quenched* when the OSC is dried to form the TFT electronic film.

In the FBI-OTFT the OSC is already deposited before the binding process and therefore its electrostatic structure is determined following the empty proteins dipole moment and cannot rearrange due to the lack of solvent molecules.

Apparently, a very week V_T_ shift is observed also when the B5F-binding is detected. This occurrence implies that the charge carried by the ligand critically matters, since only charged species are able to cause a large electrostatic rearrangement in each of the three proteins. The conformational change is therefore related to the charge carried by the biotin ligand and it is this one that generates significant charge redistribution on the protein whole structure upon binding.

The increase of the dipole moment associated to the electrostatic changes upon biotin-binding is also proved by the MD simulations whose results are shown in Fig. 4B. A priori, one would expect contribution of this dipole layer to the V_T_ shift according to the Helmholtz equation^51^:


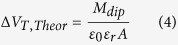


where M_dip_ is the dipole moment computed for the AV-biotin complex, ε_0_ and ε_r_ are the vacuum and the relative permittivity while A is the area of the outermost portion of the protein actually impacting on the electrostatics of the OSC 2D interfacial layer. The V_T_ shift evaluated for the AV-biotin complex according to Eq. 4 has been found to be of 23.73 V. This value is in excellent agreement with the measured V_T_ shift of 29.04 ± 5.45 V, within one standard deviation. As shown in Fig. 4D, this dipole is also found to be oriented with its positive pole pointing towards the OSC layer in correspondence with the entrance of the AV binding pocket, thus confirming the experimental evidences. Also perfectly in line with the experimental evidences is the finding that the binding to B5F does not significantly affect the dipole moment of the protein (Fig. 4B). In this case the biotin carboxylic group, responsible for the biotin negative charge, is conjugated with the fluorophore 5-fluorescein, which interacts with the lysine and tryptophan functional groups of the AV L7,8 loop, thus neutralizing the positive pole.

Another quite relevant aspect of this study involves the quantification of the μ_FET_ changes occurring upon biotin-binding to the three studied proteins. The experimental observations reported in Fig. 3D prove how a significant fractional decrease of the mobility is detected for SAV, NAV and AV proteins with both FBI and PFC-OTFT devices. However, while a consistent μ_FET_ decrease for all proteins is seen with the FBI-OTFT, much more scattered data are gathered with the PFC. Such a misalignment between the two approaches can be explained considering that the observed mobility decreases can be ascribed to the disorder induced, locally, by the proteins structural/conformational changes that impact on the OSC transport properties. This effect is clearly much more sensitively detected if the OSC is already deposited on the proteins when binding occurs. This is the case only for the FBI device. A quantitative support to the claim that disorder is induced in the P3HT by the proteins conformational changes, is provided in the following. It is well assessed that the L3,4 and L5,6 loops surrounding the biotin-binding pocket adopt a *closed* conformation upon complex formation^15^. Our MD simulations, carried out on the AV tetramer, confirm that L3,4 and L5,6 loops switch from an *open* to a *closed* conformational state in terms of a decrease in the Root Mean Square Fluctuations (RMSFs) from approximately 4 Å to 2 Å (Fig. 4C). Typically, P3HT self-assembles in lamellae whose spacing determines the transfer integral value and ultimately the charged species hopping mobility^52^. A drop of almost one order of magnitude has been computed for the transfer integral upon increase of the P3HT inter-chain distance of about 1 Å^54^. Our MD simulation quantifies the disordered induced by the fluctuation of the loops in about 2 Å of displacement, thus supporting quantitatively the claim that such a conformational change generates a local disorder on the Å length-scale that may already deeply affect the P3HT delocalization length leading to a drop in the P3HT μ_FET_ of about 20% as the protein biotin complex formation is accomplished.

The scattered mobility changes for the case of the PFC-OTFTs can be accounted for by considering that the induced electrostatic rearrangement can impact also on the mobility as this localized effect can generate a disorder at a length scale that is shorter than the OSC delocalization length.

## Conclusions

FBI and PFC-OTFTs have been demonstrated to be a powerful combined platform to quantitatively investigate protein-ligand complex formation processes, involving surface bound biological recognition elements. To this aim, a layer of SAV, NAV or AV has been integrated right at the dielectric/OSC interface of both classes devices. The protein electrostatic properties and the structural/conformational changes due to the protein-ligand complex formation have been separately addressed. This result has been achieved by decoupling the V_T_ and μ_FET_ contributions to the measured current fractional changes upon binding. Significant V_T_ fractional changes have been observed for the PFC-OTFTs upon exposure to biotin, while negligible changes have been measured for all the protein-B5F complexes in the same configuration. Biotin binding has been proved to have a negligible impact also on the V_T_ of the FBI-OTFT devices. The measured shifts in V_T_ have been related to the changes occurring in the protein dipole moment and to the OSC capability to rearrange following the new electrostatic induction. On the other hand, fractional changes of μ_FET_ have been observed only for the FBI configuration and have been related to the structural and conformational changes occurring in the protein layer upon ligand binding, directly impacting on the transport properties of the OSC.

All-atoms MD simulations have been performed on the whole AV tetramer with and without ligands, as well. MD simulations strongly support and confirm the hypotheses suggested to interpret the experimental evidences and offer valuable insights on the molecular rationale behind the observed changes in charge mobility of the OSC. This contributes to the conclusion that conformational changes occurring upon ligand binding can now be detected also by employing OTFTs. Whether the latter will serve as a tool to investigate conformational changes or, hopefully, will rely on their biological component to be embedded in point-of-care devices remains to be ascertained.

## Materials and Methods

### Materials

Streptavidin (SAV), avidin (AV), neutravidin (NAV), biotin and biotin (5 – fluorescein) conjugated (B5F) were purchased by Sigma–Aldrich and used with no further purification. Water for high performance liquid chromatography (HPLC-grade) was purchased by Sigma-Aldrich. Poly(3-hexylthiophene-2,5-diyl), P3HT (BASF, Sepiolid^TM^ P200, regioregularity >98%) was purified as reported by Urien *et al*.^55^.

### Fabrication of FBI and PFC OTFTs

All the devices were fabricated starting from a highly n-doped silicon substrate, acting as the gate contact, covered by a 300 nm thick thermally grown SiO_2_ holding a capacitance per unit area of 9 nF ∙ cm^−2^. The most relevant fabrication steps are reported in Fig. 1. The SiO_2_ surface was rinsed through a procedure involving treatment with solvents of increasing polarity. The proteins AV, SAV and NAV were purchased as powder (lyophilized from a 10 mM phosphate buffer) and were re-dissolved in water (HPLC grade) to obtain a 0.7 μM solution whose pH was 7.5. The most relevant steps in the fabrication of an FBI-OTFT are illustrated in Fig. 1A–C. 60 μL of the protein solution was then used to spin-coat the protein layer at the slow spin-rate of 200 rpm for 40 min. In the FBI-OTFT configuration the three proteins were spin-coated on different regions of the same Si/SiO_2_ substrate. The sample was then rinsed with water to remove the excess of protein. The washing water was then assayed (fluorescence) to quantify the dissolved proteins. During the washing step 47% of the protein was removed. Over the protein layer the P3HT OSC was deposited from a 2.6 mg/mL chloroform solution, by spin-coating (2000 rpm for 30 s) to cover all the three proteins regions (Fig. 1A). A raw of equally spaced source (S) and drain (D) contact pads were then patterned on the OSC by thermal evaporation (8 × 10^−7^ Torr) of gold through a shadow mask, defining channel lengths of L = 200 μm and channel widths of W = 4 mm. Each protein region hosted at least 9 different TFT channels to allow the evaluation of the error as one relative standard deviation (RSD). The device was exposed to a droplet 0.5 μL of 1 part-per-billion (1 ppb, 4.1 nM) biotin solution (Fig. 1B) attaining in the droplet area a biotin/protein molar ratio of 5 × 10^−3^. After incubation, the unreacted biotin was removed by washing with water (Fig. 1C). Notably, the porous nature of the OSC allows biotin molecules to efficiently diffuse through the OSC layer and thus reach the proteins deposited underneath^30^,^34^. The PFC-OTFT configuration fabrication steps are shown in Fig. 1E,F,C. Also in this case the biotin-binding proteins were deposited by slow spin-coating on the SiO_2_ surface (Fig. 1E). In both configurations, the proteins number is much larger than that expected for an uniform monolayer: we know from previous investigation that the deposition leads to an uniform monolayer with few islands of aggregated proteins. In this case, exposure to biotin, and eventually complex formation, was achieved before OSC deposition. The fabrication of the PFC-OTFT structure involved the deposition by slow spin-coating of the three proteins onto three different Si/SiO_2_ substrates. To this aim, 20 μL of the same protein was deposited on two different regions on each substrate. Afterwards, a 20 μL droplet of bare water and of a 1 ppb (4.1 10^−9^ molar, nM) biotin solutions were casted each on one of the two protein regions. The device was then incubated for 20 min in a humid chamber and subsequently the region exposed to the biotin solution was washed with water to remove the unreacted excess of biotin. In this case, as well, the biotin/protein molar ratio is 5 × 10^−3^.

For clearness, in Fig. 1F only the region of the protein exposed to biotin is represented. The device was then dried under a nitrogen flow and the P3HT OSC was deposited by spin-coating, as for the FBI structure, fully covering all the protein deposits. As a last step, a row of S and D contact pads was defined also for the PFC-OTFT (Fig. 1C). Also in this case each protein region hosted at least 9 different TFT channels to allow the evaluation of the error as one relative standard deviation (RSD).

### Measurement of the OTFT I–V curves

The I–V characteristics and the transfer characteristics of all the OTFTs were measured with a Keithley 4200 – SCS semiconductor parameter analyzer at room temperature and under a nitrogen flow. All the devices were tested in the common-source configuration. For the measurement of the I–V transfer curves, the gate voltage, V_G_, was cycled from the *depletion* to the *accumulation* state with pulses of alternating polarity (AP-mode)^56^. This procedure was repeated until a stable current (namely a current curve perfectly reproducing the previous trace) was measured. Specifically, a positive V_G_ pulse was followed by a negative one, both lasting 5 ms. I_DS_ was recorded before the end of each negative voltage pulse when the current reached the steady state. The application of a positive V_G_ pulse in the voltage duty-cycle has been proven to improve measurements repeatability, allowing to stabilize the device towards spurious charge trapping effects^56^,^57^. Given the measuring procedure undertaken it is unlikely that any of the V_T_ shifts measured would be determined by the presence of interfacial traps.

For the FBI configuration, the electrical measurements were carried out as follows: a 0.5 μL droplet of water (20 μL cm^−2^) was deposited directly on the OSC of one of the OTFT channel regions, namely between two contiguous S and D contacts. The water was let on the device for 10 min and dried in a nitrogen flow before measuring the output and the transfer I–V curves. This is addressed as the I_0_ current *base-line*. Afterwards, a 0.5 μL droplet of the 1 ppb biotin solution (20 μL cm^−2^) was deposited on the same channel and incubated for 10 min to allow the complex formation to be accomplished. After washing the unreacted biotin away, the device was dried under a nitrogen flow for 10 min and the transfer I–V characteristic curve was measured. This is addressed as the I *signal* current. Although care was taken to prevent wetting of the S and D pads throughout the whole measuring protocol to minimize contact resistance related effects, this was not completely unavoidable. Indeed, the I–V transfer curves of the FBI-OTFT in Fig. 2A show the current saturating at high V_G_ bias. This effect is likely due to a barrier to charges injection into the contacts that increase the contact resistance.

In the PFC-OTFT samples, the I–V output and transfer curves were measured on the TFTs patterned on the regions where the protein layer (exposed to water 60 μL cm^−2^) or the pre-formed complexes (protein layer exposed to 1 ppb of Biotin 60 μL cm^−2^) reside underneath the OSC, the former being the *base-line* the latter the *signal*. All sets of measurements (on both FBI and PFC) were performed also allowing the AV, SAV and NAV layers to interact with the B5F.

In all determinations, the error bars were taken as the relative standard deviations (RSDs) over an ensemble of measurements acquired on nine different devices (reproducibility error).

### Molecular Dynamics simulations

The initial structure of the AV-Biotin complex was obtained from the Protein Data Bank (PDB), entry 2AVI^58^. To increase the structure confidence level (to correct missing hydrogen atoms, incomplete side chains and loops, ambiguous protonation states and flipped residues) the 2AVI crystal was first pre-treated using the Schrödinger’s Protein Preparation Wizard (Version 9.5)^59^. The optimal protonation state for Histidine residues was determined according to the pH experimental value of 7.5. The simulation system was built using Visual Molecular Dynamics (VMD)^60^. Cl^−^ ions were added to neutralize the system using the VMD’s autoionize plugin. In the case of AV-Biotin complex system 4 extra Na^+^ ions were added to counterbalance the net negative charge carried by the four bounded biotins. The initial positions of the ions were taken from a previous all-atoms MD simulation performed in explicit solvent at 150 mM NaCl concentration. All modeled systems (AV, AV-biotin complex and AV-B5F complex) were simulated in vacuum in a cubic periodic box of 100 Å side generating a final system of 7524 atoms (number computed for the tetramer of the AV-biotin complex). The simulations were performed with an implicit solvent model assuming a relative permittivity ε_r_ = 3, typical for a protein system^61^. All MD simulations were performed using NAMD 2.9^59^ and the CHARMM36^62^ force-field. Atomic charges of biotin and B5F were determined by the Restrained Electrostatic Potential (RESP)^63^ method using Hartree-Fock theory and the 6-31G**basis set. The full systems were minimized to remove steric clashes in the initial geometry and gradually heated up to 297 K within 500 ps of MD. The SHAKE algorithm was employed to constrain all R–H bonds. Periodic boundary conditions were applied in all directions. A non-bonded cut-off of 12 Å was used, whereas the Particle-Mesh-Ewald (PME) was employed to include the contributions of long-range interactions. All simulations were performed in an isothermal-isobaric ensemble (1 atm, 297 K) with a Nosè–Hoover Langevin barostat^62^,^64^ (oscillation period 200 fs, decay coefficient 100 fs) and a Langevin thermostat^65^ (damping coefficient 1 ps^−1^). The time step was set to 2 fs, and coordinates were saved every 5000 steps (10 ps). For all the considered systems, a MD trajectory of 50 ns was obtained and since the equilibration of the structure required less than 5 ns, the first 5 ns were removed from the analysis.

The electric dipole moment was calculated for each monomer (including the ligand when present) as the modulus of:


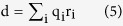


where *i* runs over the atoms of the monomer (monomer-ligand complex), q_*i*_ is the partial charge of the i-th atom and r_*i*_ is the position vector of the i-th atom respect to the center of mass of the monomer (monomer-ligand complex). Root Mean Square Deviations (RMSDs) and Root Mean Square Fluctuations (RMSFs) were obtained after alignment of the trajectory to all the C-alpha atoms belonging to the monomer under investigation^66^,^67^,^68^,^69^,^70^,^71^,^72^,^73^,^74^,^75^

## Additional Information

**How to cite this article**: Macchia, E. *et al*. Organic bioelectronics probing conformational changes in surface confined proteins. *Sci. Rep.* **6**, 28085; doi: 10.1038/srep28085 (2016).

## References

[b1] Grange R. D., Thompson J. P. & Lambert D. G. Radioimmunoassay, enzyme and non-enzyme-based immunoassays. Br. J. Anaesth. 112, 213–216 (2014). 24431350 10.1093/bja/aet293

[b2] Homola J. Surface Plasmon Resonance Sensors for Detection of Chemical and Biological Species. Chem. Rev. 108(2), 462–493 (2008). 18229953 10.1021/cr068107d

[b3] Buttry D. A. & Ward M. D. Measurement of interfacial processes at electrode surfaces with the electrochemical quartz crystal microbalance. Chem. Rev. 92(6), 1355–1379 (1992).

[b4] Casalini S. . Multiscale Sensing of Antibody–Antigen Interactions by Organic Transistors and Single-Molecule Force Spectroscopy. ACS Nano 9, 5051 (2015). 25868724 10.1021/acsnano.5b00136

[b5] Duan X., Fu T. M., Liu J. & Lieber C. M. Nanoelectronics-biology frontier: From nanoscopic probes for action potential recording in live cells to three-dimensional cyborg tissues. Nano Today 8(4), 351–373 (2013). 24073014 10.1016/j.nantod.2013.05.001 PMC3781175

[b6] Chiti F. & Dobson C. M. Protein misfolding, functional amyloid, and human disease. Annu. Rev. Biochem. 75, 333–366 (2006). 16756495 10.1146/annurev.biochem.75.101304.123901

[b7] Peterson J. I. & Vurek G. G. Fiber-optic sensors for biomedical applications. Science 224, 123–127 (1984). 6422554 10.1126/science.6422554

[b8] Rivnay J., Owens R. M. & Malliaras G. G. The Rise of Organic Bioelectronics. Chem. Mater. 26(1), 679–685 (2014).

[b9] Magliulo M., Manoli K., Palazzo G. & Torsi L., Organic field-effect transistor sensors: a tutorial review. Chem Soc Rev. 42(22), 8612–8628 (2013). 24018860 10.1039/c3cs60127g

[b10] Manoli K. . Printable Bioelectronics to Investigate Functional Biological Interfaces. Angew. Chem. Int. Ed. 54, 2–17 (2015). 10.1002/anie.201502615 26420480

[b11] Lehnert M. . Adsorption and Conformation Behavior of Biotinylated Fibronectin on Streptavidin-Modified TiO_X_ Surfaces Studied by SPR and AFM. Langmuir 27, 7743–7751 (2011). 21598954 10.1021/la200908h

[b12] De Chancie J. & Houk K. N. The Origins of Femtomolar Protein−Ligand Binding: Hydrogen-Bond Cooperativity and Desolvation Energetics in the Biotin−(Strept)Avidin Binding Site. J. Am. Chem. Soc. 129, 5419–5429 (2007). 17417839 10.1021/ja066950n PMC2527462

[b13] Nguyen T. T., Sly K. L. & Conboy J. C. Comparison of the Energetics of Avidin, Streptavidin, NeutrAvidin, and Anti-Biotin Antibody Binding to Biotinylated Lipid Bilayer Examined by Second-Harmonic Generation. Anal. Chem. 84, 201–208 (2012). 22122646 10.1021/ac202375n

[b14] Gonzàlez M. . Interaction of biotin with streptavidin. Thermostability and conformational changes upon binding. J. Biol. Chem. 272, 11288–11294 (1997). 9111033 10.1074/jbc.272.17.11288

[b15] Lefèvre T. & Subirade M. Molecular structure and interaction of biopolymers as viewed by Fourier transform infrared spectroscopy: model studies on beta-lactoglobulin. Food Hydrocolloids 15, 365–376 (2001).

[b16] Freitag S., Le Trong I., Klumb L., Stayton P. S. & Stenkamp R. E. Structural studies of the streptavidin binding loop. Protein Science 6, 1157–1166 (1997). 9194176 10.1002/pro.5560060604 PMC2143724

[b17] Katz B. A. Binding of biotin to streptavidin stabilizes intersubunit salt bridges between Asp61 and His87 at low pH. J. Mol. Biol. 274, 776–800 (1997). 9405158 10.1006/jmbi.1997.1444

[b18] Rosano C., Arosio P. & Bolognesi M. The X-ray three-dimensional structure of avidin. Biomolecular Engineering 16, 5–12 (1999). 10796979 10.1016/s1050-3862(99)00047-9

[b19] Weber P. C., Wendolski J. J., Pantoliano M. W. & Salemme F. R. J. Crystallographic and thermodynamic comparison of natural synthetic ligands bound to streptavidin. Am. Chem. Soc. 114, 3197−3200 (1992).

[b20] Jones M. L. & Kurzban G. P. Noncooperativity of Biotin Binding to Tetrameric Streptavidin. Biochemistry 34, 11750–11756 (1995). 7547907 10.1021/bi00037a012

[b21] Gonzales M., Argarana C. E. & Fidelio G. D. Extremely high thermal stability of streptavidin and avidin upon biotin binding. Biomolecular Engineering 16, 67–72 (1999). 10796986 10.1016/s1050-3862(99)00041-8

[b22] Cerutti D. S., Trong I. L., Stenkamp R. E. & Lybrand T. P. J. Dynamics of the streptavidin-biotin complex in solution and in its crystal lattice: Distinct behavior revealed by molecular simulations. Phys. Chem. B 113, 6971−6985 (2009). 10.1021/jp9010372 PMC2791092 19374419

[b23] Tong Y., Mei Y., Li Y. L., Ji C. G. & Zhang J. Z. H. Electrostatic Polarization Makes a Substantial Contribution to the Free Energy of Avidin−Biotin Binding. J. Am. Chem. Soc. 132, 5137–5142 (2010). 20302307 10.1021/ja909575j

[b24] Bykhovski A., Zhang W., Jensen J. & Woolard D. Analysis of electronic structure, binding, and vibrations in biotin-streptavidin complexes based on density functional theory and molecular mechanics. J. Phys. Chem. B 117, 25−37 (2013). 23214720 10.1021/jp3075833

[b25] Kneipp K., Kneipp H., Itzkan I., Dasari R. R. & Feld M. S. Surface-enhanced Raman scattering and biophysics. J. Phys. Condens. Matter 14, R597–R624 (2002).

[b26] Stewart S. & Fredericks P. M. Surface-enhanced Raman spectroscopy of peptides and proteins adsorbed on an electrochemically prepared silver surface. Spectrochimica Acta Part A 55, 1615–1640 (1999).

[b27] Homola J. Surface plasmon resonance sensors for detection of chemical and biological species, Chem. Rev. 108, 462–493 (2008). 18229953 10.1021/cr068107d

[b28] Stiles P. L., Dieringer J. A., Shah N. C. & Van Duyne R. P. Surface-enhanced Raman spectroscopy. Annu. Rev. Anal. Chem. 1, 601 (2008). 10.1146/annurev.anchem.1.031207.112814 20636091

[b29] Grubmuller H., Heymann B. & Tavan P. Ligand Binding: Molecular Mechanics Calculation of the Streptavidin-Biotin Rupture Force. Science 271, 997–999 (1996). 8584939 10.1126/science.271.5251.997

[b30] Yuan C., Chen A., Kolb P. & Moy V. Y. Energy Landscape of Streptavidin−Biotin Complexes Measured by Atomic Force Microscopy, Biochemistry. 39, 10219–10223 (2000). 10956011 10.1021/bi992715o

[b31] Herne T. M., Ahern A. M. & Garrell R. L. Surface-enhanced Raman spectroscopy of tripeptides adsorbed on colloidal silver. Anal. Chim. Acta 246, 75–84 (1991).

[b32] Seiki N. . Rational synthesis of organic thin films with exceptional long-range structural integrity. Science 348, 1122–1126 (2015). 26045433 10.1126/science.aab1391

[b33] Matsuhisa N. . Printable elastic conductors with a high conductivity for electronic textile applications. Nature Commun. 6, 7461 (2015). 26109453 10.1038/ncomms8461 PMC4491189

[b34] Stoliar P. . DNA adsorption measured with ultra-thin film organic field effect transistors. Biosens. Bioelectron. 24, 2935–2938 (2009). 19272764 10.1016/j.bios.2009.02.003

[b35] Spanu A. . An organic transistor-based system for reference-less electrophysiological monitoring of excitable cells. Sci. Rep. 5, 8807 (2015). 25744085 10.1038/srep08807 PMC4351515

[b36] Hammock M. L., Knopfmacher O., Naab B. D., Tok J. B.-H. & Bao Z. Investigation of Protein Detection Parameters Using Nanofunctionalized Organic Field-Effect Transistors. ACS Nano 7, 3970–3980 (2013). 23597051 10.1021/nn305903q

[b37] Magliulo M. . Structural and Morphological Study of a Poly(3-hexylthiophene)/Streptavidin Multilayer Structure Serving as Active Layer in Ultra-Sensitive OFET Biosensors. J. Phys. Chem C 118, 15853–15862 (2014).

[b38] Torsi L., Dodabalapur A., Sabbatini L. & Zambonin P. G. Multi-parameter gas sensors based on organic thin-film-transistors. *Sens*. Actu. B 67(3), 312–316 (2000).

[b39] Mulla M. Y. . Capacitance-modulated transistor detects odorant binding protein chiral interactions. Nature Commun. 6, 6010 (2015). 25591754 10.1038/ncomms7010 PMC4309438

[b40] Yang S. Y. . Electrochemical Transistors with Ionic Liquids for Enzymatic Sensing. Chem. Commun. 46, 7972–7974 (2010). 10.1039/c0cc02064h 20871879

[b41] Kergoat L. . DNA detection with a water-gated organic field-effect transistor. Org. Electron. 13, 1–6 (2012).

[b42] Magliulo M., Manoli K., Macchia E., Palazzo G. & Torsi L. Tailoring Functional Interlayers in Organic Field-Effect Transistor Biosensors. Adv. Mater. 10.1002/adma.201403477 (2014). 25429859

[b43] Angione M. D. . Interfacial electronic effects in functional biolayers integrated into organic field-effect transistors. PNAS 109(17), 6429–6434 (2012). 22493224 10.1073/pnas.1200549109 PMC3340085

[b44] Sze S. M. & Ng K. K. Physics of semiconductor devices (John Wiley & Sons, ed. 3, 2006).

[b45] Macchia E., Giordano F., Magliulo M., Palazzo G. & Torsi L. An analytical model for bio-electronic organic field-effect transistor sensors, Appl. Phys. Lett. 103, 103301 (2013).

[b46] Kergoat L. . A Water-Gate Organic Field-Effect Transistor. Adv. Mater. 22, 2565 (2010). 20491093 10.1002/adma.200904163

[b47] Izrailev S., Stepaniants S., Balsera M., Oono Y. & Schulten K. Molecular dynamics study of unbinding of the avidin-biotin complex. Biophys. J. 72(4), 1568–1581 (1997). 9083662 10.1016/S0006-3495(97)78804-0 PMC1184352

[b48] Dodabalapur A., Torsi L. & Katz H. Organic Transistors: Two-Dimensional Transport and Improved Electrical Characteristics. Science 268, 270–271 (1995). 17814790 10.1126/science.268.5208.270

[b49] London F. The general theory of molecular forces. Trans. Faraday Soc. 33, 8b–26 (1937).

[b50] Pernstich K. P. . Threshold voltage shift in organic field effect transistors by dipole monolayers on the gate insulator. J. Appl. Phys. 96, 6431–6438 (2004).

[b51] Possaner S. K., Zojer K., Pacher P., Zojer E. & Schurrer F. Threshold Voltage Shifts in Organic Thin-Film Transistors Due to Self-Assembled Monolayers at the Dielectric Surface. Adv. Funct. Mater. 19, 958–967 (2009).

[b52] Dezieck A. . Threshold voltage control in organic thin film transistors with dielectric layer modified by a genetically engineered polypeptide. Appl. Phys. Lett. 97, 013307 (2010).

[b53] Alberga D., Mangiatordi G. F., Torsi L. & Lattanzi G. Effects of Annealing and Residual Solvents on Amorphous P3HT and PBTTT Films. J. Phys. Chem. C 118, 8641–8655 (2014).

[b54] Lan Y. K. & Huang C. I. A. Theoretical Study of the Charge Transfer Behavior of the Highly Regioregular Poly-3-hexylthiophene in the Ordered State. J. Phys. Chem. B 112, 14857–14862 (2008). 18973359 10.1021/jp806967x

[b55] Urien W. . Field-effect transistors based on poly(3-hexylthiophene): Effect of impurities. Org. Electron. 8(6), 727–734 (2007).

[b56] Tero R., Watanabe H. & Urisu T. Supported phospholipid bilayer formation on hydrophilicity-controlled silicon dioxide surfaces. Phys. Chem. Chem. Phys. 8(33), 3885–3894 (2006). 19817049 10.1039/b606052h

[b57] Crone B. . Electronic sensing of vapors with organic transistors. Apl. Phys. Lett. 78, 2229 (2001).

[b58] Cotrone S. . Microcantilevers and organic transistors: two promising classes of label-free biosensing devices which can be integrated in electronic circuits. Anal Bioanal Chem. 403(1), 1799–1811 (2012). 22189629 10.1007/s00216-011-5610-2 PMC7079887

[b59] Livnah O., Bayer E. A., Wilchek M. & Sussman J. L. Three-dimensional structures of avidin and the avidin-biotin complex. PNAS 90(11), 5076–5080 (1993). 8506353 10.1073/pnas.90.11.5076 PMC46657

[b60] Schrodinger Release 2013-2, Maestro, version 9.5, Schrodinger, LLC, New York, NY, (2013).

[b61] Kada G., Falk H. & Gruber H. J. Accurate measurement of avidin and streptavidin in crude biofluids with a new, optimized biotin–fluorescein conjugate. Biochimica et Biophysica Acta (BBA)-General Subjects 1427(1), 33–43 (1999). 10082985 10.1016/s0304-4165(98)00178-0

[b62] Kukic P. . Protein Dielectric Constants Determined from NMR Chemical Shift Perturbations, J. Am. Chem. Soc. 135(45), 16968–16976 (2013). 24124752 10.1021/ja406995j PMC4480918

[b63] Phillips C. . Scalable molecular dynamics with NAMD. J. Comput. Chem. 26, 1781–1802 (2005). 16222654 10.1002/jcc.20289 PMC2486339

[b64] MacKerell A. D., Banavali N. & Foloppe N. Development and current status of the CHARMM force field for nucleic acids. Biopolymers 56, 257–265 (2000). 11754339 10.1002/1097-0282(2000)56:4<257::AID-BIP10029>3.0.CO;2-W

[b65] Feller S. E., Zhang Y., Pastor R. W. & Brooks B. R. Constant pressure molecular dynamics simulation: The Langevin piston method. J. Chem. Phys. 103, 4613–4621 (1995).

[b66] Green N. M. Avidin and streptavidin. Methods Enzymol. 184, 51–67 (1990). 2388586 10.1016/0076-6879(90)84259-j

[b67] Magliulo M. . Part per Trillion Label-Free Electronic Bioanalytical Detection. Anal. Chem. 85(8), 3849–3857 (2013). 23323705 10.1021/ac302702n

[b68] Adelman S. A. & Doll J. D. Generalized Langevin equation approach for atom/solid‐surface scattering: General formulation for classical scattering off harmonic solids. J.Chem. Phys. 64, 2375–2388 (1976).

[b69] Riepl M. . Optimization of capacitive affinity sensors: drift suppression and signal amplification. Anal. Chim. Acta 392, 77–84 (1999).

[b70] Ishikawa F. N. . A Calibration Method for Nanowire Biosensors to Suppress Device-to-Device Variation. ACS Nano 3, 3969–3976 (2009). 19921812 10.1021/nn9011384 PMC2805439

[b71] Bredas J. L., Beljonne D., Coropceanu V. & Cornil J., Charge-Transfer and Energy-Transfer Processes in π-Conjugated Oligomers and Polymers: A Molecular Picture. Chem. Rev. 104, 4971–5003 (2004). 15535639 10.1021/cr040084k

[b72] Manoli K. . Pulsed voltage driven organic field-effect transistors for high stability transient current measurements. Org. Electron. 15(10), 2372–2380 (2014).

[b73] Humphrey W., Dalke A. & Schulten K. VMD: visual molecular dynamics. J. Mol. Graph. 14(33–38), 27–28 (1996). 10.1016/0263-7855(96)00018-5 8744570

[b74] Bayly C. I., Cieplak P., Cornell W. D. & Kollman P. A., A well-behaved electrostatic potential based method using charge restraints for deriving atomic charges: the RESP model. J. Phys. Chem. 97, 10269–10280 (1993).

[b75] Martyna G. J., Tobias D. J. & Klein M. L. Constant pressure molecular dynamics algorithms. J. Chem. Phys. 101, 4177–4189 (1994).

